# Effect of the consumption of a new symbiotic shake on glycemia and cholesterol levels in elderly people with type 2 diabetes mellitus

**DOI:** 10.1186/1476-511X-11-29

**Published:** 2012-02-22

**Authors:** Camila Moroti, Loyanne Francine Souza Magri, Marcela de Rezende Costa, Daniela CU Cavallini, Katia Sivieri

**Affiliations:** 1 Department of Food & Nutrition, School of Pharmaceutical Sciences, São Paulo State University, Araraquara, SP, Brazil; 2 Paulista Júlio de Mesquita Filho, Faculdade de Ciências Farmacêuticas, Departamento de Alimentos e Nutrição, Universidade Estadual, Rodovia Araraquara Jaú, Km 1, Campus Universitário, Caixa Postal 502, CEP 14802901 Araraquara, SP, Brazil

**Keywords:** *Bifidobacterium bifidum*, Fructooligosaccharide, *Lactobacillus acidophilus*, Symbiotics, *Shake*

## Abstract

**Background:**

The consumption of foods containing probiotic and prebiotic ingredients is growing consistently every year, and in view of the limited number of studies investigating their effect in the elderly.

**Objective:**

The objective of this study was to evaluate the effect of the consumption of a symbiotic shake containing *Lactobacillus acidophilus, Bifidobacterium bifidum *and fructooligosaccharides on glycemia and cholesterol levels in elderly people.

**Methods:**

A randomized, double-blind, placebo-controlled study was conducted on twenty volunteers (ten for placebo group and ten for symbiotic group), aged 50 to 60 years. The criteria for inclusion in the study were: total cholesterol > 200 mg/dL; triglycerides > 200 mg/dL and glycemia > 110 mg/dL. Over a total test period of 30 days, 10 individuals (the symbiotic group) consumed a daily dose of 200 mL of a symbiotic shake containing 10^8 ^UFC/mL *Lactobacillus acidophilus*, 10^8 ^UFC/mL *Bifidobacterium bifidum *and 2 g oligofructose, while 10 other volunteers (the placebo group) drank daily the same amount of a shake that did not contain any symbiotic bacteria. Blood samples were collected 15 days prior to the start of the experiment and at 10-day intervals after the beginning of the shake intake. The standard lipid profile (total cholesterol, triglycerides and HDL cholesterol) and glycemia, or blood sugar levels, were evaluated by an enzyme colorimetric assay.

**Results:**

The results of the symbiotic group showed a non-significant reduction (*P *> 0.05) in total cholesterol and triglycerides, a significant increase (*P *< 0.05) in HDL cholesterol and a significant reduction (*P *< 0.05) in fasting glycemia. No significant changes were observed in the placebo group.

**Conclusion:**

The consumption of symbiotic shake resulted in a significant increase in HDL and a significant decrease of glycemia.

**Trial Registration:**

ClinicalTrials.gov: NCT00123456

## Background

At present, cardiovascular diseases are among the major causes of death in the world. In Brazil, cholesterol is responsible for nearly 8% of the deaths caused by non-transmissible diseases, which represents around 4.5 million people. On the other hand, lipid abnormalities associated with increased glycemia, or even diabetes, are dangerous in view of the high vulnerability of these volunteers to coronary diseases [1]. Epidemiological studies show a positive relation between increased blood sugar levels (glycemia), lipid dysfunction and cardiovascular diseases [2].

Cardiovascular disease (CVD) is the primary cause of death in people with type 2 diabetes mellitus (T2DM). Relative risk of CVD is 2- to 4-fold higher in diabetic people compared with nondiabetic people [3]. Dyslipidemia has been identified as a risk factor for cardiovascular complications in T2DM. The American Diabetes Association guidelines indicate that, the primary goal of low-density lipoprotein cholesterol (LDL-C) concentration is less than 2.6 mmol/L (100 mg/dL) in diabetic people without overt CVD [4].

Probiotic agents are defined as live microorganisms that when administered in adequate amounts confer health benefits on the host [5].There are some reports in literature suggesting or demonstrating that the consumption of food products containing probiotic microorganisms may reduce the level of serum cholesterol in humans [6,7]. The first scientific report associating lactic acid bacteria with a reduction in serum cholesterol was published in 1974 by Mann and Spoerry [8], who observed an 18% reduction in the total serum cholesterol level of warriors of an African tribe whose regular diet included milk fermented by lactobacillus.

Prebiotics can be defined as non-digestible food ingredients that beneficially affect the host by stimulating the growth and/or activity of one or a limited number of desired bacteria in the colon, such as bifidobacteria, which are regarded as beneficial to human health. Although prebiotic components primarily act in the large intestine, they also may have some impact on microorganisms in the small intestine [9].

The prebiotics identified thus far are non-digestible carbohydrates, including lactulose, inulin and several oligosaccharides that provide carbohydrates which the colon's beneficial bacteria are able to ferment [10]. Some of the effects attributed to prebiotics include the modulation of key physiological functions, such as the absorption of calcium, lipid metabolism, glycemia, modulation of the composition of the intestinal microbiota - which plays a primary role in gastrointestinal physiology, and a reduced risk of colon cancer [11]. There are some reports linking the consumption of prebiotics to reduced glycemia [10]. The combination of the probiotic and prebiotic might improve the survival of the bacteria crossing the upper part of the gastrointestinal tract, thereby enhancing their effects in the large bowel. In addition, their effects might be additive or even synergistic [10,11].

As the consumption of foods containing probiotic and prebiotic ingredients is growing consistently every year, and in view of the limited number of studies investigating their effect in the elderly, the objective of this study was to evaluate the effect of the consumption of a symbiotic shake containing *Lactobacillus acidophilus, Bifidobacterium bifidum *and fructooligosaccharides on glycemia and cholesterol levels in elderly people.

## Materials and methods

### Production of the symbiotic shake and the placebo shake

The symbiotic shake was developed and manufactured by the company Maxinutri Alimentos LTDA from a formulation containing 9% skim milk powder (Confepar), 23% whey powder (Alibra), 21% maltodextrin (Companhia Lorenz), 15% oatmeal (SL Alimentos), 7% texturized soybean protein TSP (Texpro), 5% soybean fiber (Gama AS), 3.5% guar gum (Doce Aroma), 3.5% collagen (Gelita do Brasil), 5% soybean extract (San Luca), 4.5% fructooligosaccharide (Corns Products), 0.032% sucralose (China), 0.024% acessulfame (China), 0.7% tricalcium phospfate (China), 2% *Lactobacillus acidophilus *(Fortitech), 2% *Bifidobacterium bifidum *(Fortitech), 1.55% flavor (Doce aroma), 0.035% sodium chloride (China) and 0.032 caramel color (Corns Products).

All ingredients were added to a Ribbon paddle blender. In a first stage, a premix was prepared from the ingredients of greater volume, which individually represent more than 3% of the total volume of the formulation. The remaining ingredients were added in small amounts in a way so as to obtain a homogeneous and uniform blend. The final blend was filled into plastic containers sealed with aluminum foil and labeled with information regarding the date of manufacture, expiry date and instructions on how to prepare the product for consumption. The placebo shake was manufactured the same way as the symbiotic dry mix, but without the addition of the probiotics *Lactobacillus acidophilus *and *Bifidobacterium bifidum*, nor the prebiotic fructooligosaccharide.

### Selection of volunteers

The study was conducted with healthy female individuals, aged 50-65 years, all living in Arapongas located in the northern part of the State of Paraná, Brazil. The criteria for inclusion in the study were: total cholesterol > 200 mg/dL; triglycerides > 150 mg/dL and glycemia > 110 mg/dL. All volunteers were taking glucose-lowering medications however, drug treatment was not able to normalize blood glucose levels in these volunteers. None were using hypolipidemic agents, antibiotics and minerals or vitamins supplements.

The selected individuals signed a free informed consent form and were free to withdraw at any time of the experimental period at their own discretion. The volunteers were fully informed about the type and purpose of the study, the expected conduct and all possible risks, reinforcing the importance of not skipping doses or discontinuing the medication in the course of the experiment. Written informed consent was obtained from the participants, and the study protocol was approved by the Ethics in Research Committee of the Universidade Norte do Paraná **(Protocol PP 0186/09)**.

Clinical characteristics at the baseline of the volunteers are presented in Table 1.

**Table 1:** Clinical characteristics at baseline of the volunteers

Clinical characteristics	GS (n = 9)	GP (n = 9)
Age (yr)	55.47^a ^± 2.0	56,89 ^a ^± 1.7

Height (m)	1.65 ^a ^± 1.2	1.64 ^a ^± 1.3

Weight (Kg)	75.43 ^a ^± 2.01	75.89 ^a ^± 2.00

BMI (Kg/m^2^)*	27.70 ^a ^± 0.78	28.21 ^a ^± 0.85

Diabetes	9	9

Hypertension	7	8

Smoking	5	4

Energy (kcal/day)	2144.6 ^a ^± 643.5	2322.4 ^a ^± 543.5

Values are mean ± SD. Statistical comparison of groups: means with identical minuscule letters in the same line do not differ significantly (*P *≤ 0.05). *GS *group symbiotic shake, *GP *group placebo shake, *BMI body massindex

### Study design and intervention

A 45-day, double-blind, placebo-controlled, randomized study was conducted on twenty volunteers divided into two groups (n = 10): Group S (individuals who consumed the symbiotic shake) and Group P (individuals who consumed the placebo shake) (NCT00123456; http://www.clinicaltrials.gov).

**Figure 1:**
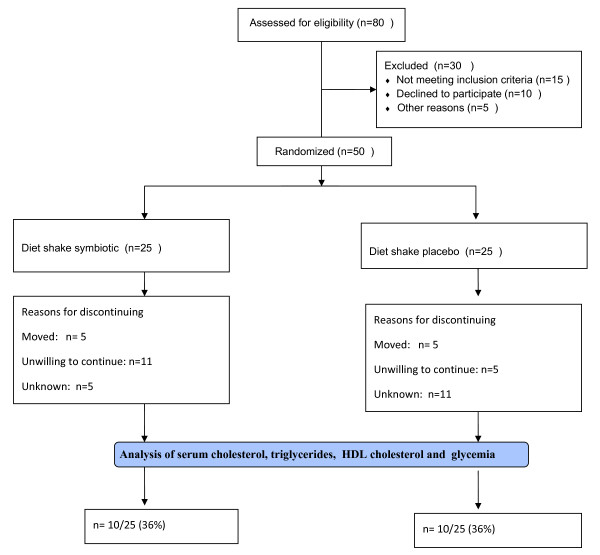
**Study design**.

The randomization (Figure 1) was conducted and the list generated by a statistician (TP) not involved in recruitment or study visits, according to computer-generated block randomization of 10 women to receive shake with probiotic or shake with placebo. The study was divided into two periods; pre-ingestion, which volunteers not ingested symbiotic or placebo product during 15 days and ingested period, which volunteers ingested during 30 days the symbiotic or placebo product. During the ingestion period the volunteers of one group (group S) consumed 2 daily doses of 100 mL symbiotic shake containing 4 × 10^8 ^UFC/100 mL *Lactobacillus acidophillus*, 4 × 10^8 ^UFC/100 mL *Bifidobacterium bifidum *and 1 g/100 mL of fructooligosaccharides. The other group (group P) consumed 2 daily doses of 100 mL shake without added probiotics and prebiotic. The volunteers were instructed to maintain their eating habits and physical activity during the study (pre-ingestion - 15 days; ingestion period - 30 days). Both groups consumed their respective dietary supplement shake for a total period of 30 days.

The products were freshly produced every week and delivered to each volunteer in plastic vials labeled with the date of manufacture, expiry date and instructions for preparation and consumption.

### Collection of blood samples

At the end of the pre-ingestion period and at every 10 days of the ingestion period the following blood samples were taken from the volunteers: 1 sample of blood containing anticoagulant (fluoride) for the determination of glycemia and another blood sample without anticoagulant for the determination of cholesterol. The volunteers were asked to fast for at least 8 hours before the blood draw to prevent possible interference with postprandial lipemia which in common in non-fasting blood samples. The plasma was separated from formed elements of the blood immediately after collection by centrifugation at 2500 rpm for 10 minutes. Serum was collected after allowing for clot formation at 37°C in a water bath and subsequent centrifugation at 2500 rpm for 10 minutes. Both serum and plasma were kept at normal refrigerator temperature (2 to 8°C) until analysis [12].

To ensure efficient and reliable performance in analyzing the biochemical parameters, an aliquot of control serum (Lot number: 40380801/2) - supplied by the Brazilian National Program for Quality Control (Programa Nacional de Controle de Qualidade PNCQ - SBAC) - was processed along with the experimental samples at each test run.

### Analysis of serum lipids

Serum total cholesterol and triglycerides levels in the volunteers blood samples were determined by an enzyme-colorimetric method (Bio Diagnóstica) using a semi-automatic apparatus (Bio-plus).

HDL cholesterol was separated based on the selectivity of the phosphotungstate and magnesium ions which promote selective precipitation of all lipoproteins (kilomicrons, VLDL and LDL), except for the HDL fraction. After centrifugation, the HDL cholesterol contained in the supernatant was determined by the same method used for total cholesterol.

Non-HDL cholesterol was calculated by subtracting HDL-C from TC and was composed of the LDL + IDL + VLDL cholesterol fractions.

### Analysis of glycemia

Fasting glycemia was determined in the fluoride plasma of the volunteers by an enzyme-colorimetric method (Bio Diagnóstica). Glucose oxidase (GOD) catalyzes the oxidation of glucose to gluconic acid and hydrogen peroxidase. Through a peroxidase-catalyzed oxidative coupling reaction (POD), the thus formed hydrogen peroxide reacts with 4-aminoantipyrin and phenol, forming a red-colored complex (quinoneimine), the absorbance of which measured at 500 nm is directly proportional to the glucose concentration in the blood sample. Absorbance was determined using a semi-automated apparatus (Bio-plus), which provides a reading of the glucose concentration expressed as mg/dL plasma.

### Statistical analyses

The results were analyzed by analysis of variance (ANOVA) and Tukey's means comparison test using the STATISTICA 6.0 software program. The differences were considered significant at the *P *< 0.05 level of confidence. All data are presented as a mean ± SD.

## Results

### Lipid profile

Table 2 depict the results of the lipid profile of the S Group (individuals who consumed the symbiotic shake) and the P Group (individuals who consumed a placebo shake) broken down over four distinct periods T0 (pre-ingestion), T1 (10th day of ingestion), T2 (20th day of ingestion) and T3 (30th day of ingestion).

**Table 2:** Lipid profile of the symbiotic and placebo groups during pre-ingestion and ingestion of the symbiotic shake

Parameter	T0	T1	T2	T3	Variation in the period T0-T3 (%)
**Total Cholesterol (mg/dL)**					

Group S	229.67 ± 55.51 ^a^	175.33 ± 49.09 ^b^	188.22 ± 29.03 ^ab^	170.33 ± 28.85 ^b^	-25.84% (p < 0.05)

Group P	204.00 ± 3.97 ^a^	160.56 ± 19.94 ^a^	194.67 ± 49.75 ^a^	191.44 ± 33.78 ^a^	-4.69% (p > 0.05)

**HDL-C (mg/dL)**					

Group S	43.33 ± 12,94 ^a^	48.78 ± 13,10 ^a^	51.11 ± 15.16 ^a^	58.56 ± 11.08 ^a^	+35.15% (p > 0.05)

Group P	50.22 ± 18.30 ^a^	49.22 ± 14.67 ^a^	49.33 ± 18.72 ^a^	55.11 ± 14.34 ^a^	+9.74% (p > 0.05)

**nHDL-C (mg/dL)**					

Group S	190.44 ± 58.19 ^a^	126.56 ± 43.00 ^b^	137.11 ± 30.39 ^b^	111.78 ± 24.68 ^b^	-41,30 (p < 0.05)

Group P	153.78 ± 18.21 ^a^	111.33 ± 29.06 ^a^	145.33 ± 59.96 ^a^	136.33 ± 32.79 ^a^	-11.35 (p > 0.05)

**Triglycerides (mg/dL)**					

Group S	317.56 ± 101.08 ^a^	248.89 ± 62.95 ^ab^	204.00 ± 89,90 ^b^	199.22 ± 54,60 ^b^	-37.27% (p < 0.05)

Group P	239.44 ± 58.38 ^a^	217.33 ± 56.47 ^a^	226.33 ± 126.23 ^a^	198.56 ± 66.07 ^a^	-17.07% (p > 0.05)

Means followed by the same lower case letters in the same line are not statistically different within the same group (*P *< 0.05)

T0: Pre-ingestion period, T1: 10th day of ingestion, T2: 20th day of ingestion, T3: 30th day of ingestion

Group S: Individuals who consumed the symbiotic shake

Group P: Individuals who consumed the placebo shake

The data contained in Table 2 show that after 4 weeks of treatment, individuals that consumed the symbiotic shake presented a reduction of 25.84% and 37.27% in their serum total cholesterol and triglyceride levels, respectively.

Three volunteers had levels of triglycerides above 400 mg/dl and therefore, we chose to calculate the concentration of nHDL-C, which represents the potentially atherogenic particles (LDL, IDL, VLDL) [13]. At the end of the experiment the volunteers that ingested the symbiotic shake showed a significant reduction on n-HDL-C, compared with the pre-treatment time (41.30%; *p *< 0.05).

With regard to HDL cholesterol (High density lipoprotein) of the S and P groups over the experimental period, a significant increase (35.15%; *p *< 0.05) was observed in the group that consumed the symbiotic shake (43.33 to 58.56 mg/dL).

The group that took the placebo shake over the same period of time did not exhibit any significant change (*p *> 0.05) relative to the total cholesterol, HDL-C; n-HDL-C and triglycerides concentration (4.69, 9.74, 11.35, 17.07%, respectively).

### Glycemia

Table 3 presents the levels of fasting glycemia levels for the individuals that participated in the study. A significant reduction (38.89%, *p *< 0.05) in glycemia (191.11 to 116.78 mg/dL) was observed over the course of the study in the individuals who consumed the symbiotic shake. However, no significant change (*p *> 0.05) was noted in the glycemia levels of the indivuals who consumed the placebo shake.

**Table 3:** Effect of the consumption of a symbiotic shake and a placebo shake on glycemia (mg/dL)

Period	Group S	Group P
**T0**	191.11 ± 18.31 ^a^	136.78 ± 19.47 ^a^

**T1**	128.56 ± 29.23 ^b^	121.33 ± 1.00 ^a^

**T2**	123.22 ± 27.78 ^b^	110.11 ± 19.90 ^a^

**T3**	116.78 ± 18.96 ^b^	110.56 ± 29.90 ^a^

Means followed by the same lower case letters in the same column are not statistically different within the same group (*P *< 0.05)

T0: Pre-ingestion period, T1: 10th day of ingestion, T2: 20th day of ingestion, T3: 30th day of ingestion

Group S: Individuals who consumed the symbiotic shake

Group P: Individuals who consumed the placebo shake

## Discussion

Changes in lifestyle, including a balanced diet and regular physical exercise may be the first step towards reducing LDL cholesterol and glycemia levels [14]. As for eating habits in particular, the consumption of fermented foods, fibers and oligosaccharides may contribute to reducing cholesterol and glycemia [15]. Probiotic bacteria and prebiotics have been introduced into the gastrointestinal tract through food matrices such as yogurts and cheeses [10]. However, at present, efforts are directed towards developing other, non-dairy foods to be used as food matrix to deliver probiotics to the intestinal tract, including shake [16] and cereal bars [17], among others. There are several studies on the efficacy of the consumption of probiotics on the reduction of blood cholesterol levels [15,18], with some of these also reporting reduced glycemia as a result of the regular intake of prebiotics [19,20]. However, the literature contains only few and fragmentary data and information on the effect of probiotics and prebiotics in elderly people. For that reason, the purpose of the present study was to investigate and evaluate the effect of the consumption of a symbiotic shake on the lipid profile and glycemia in elderly people. To this purpose, two periods were evaluated: (1) a pre-ingestion period (15 days prior to initiating the consumption of the products); and (2) the ingestion period (30 days of continued consumption of the products).

At the end of the ingestion period, the volunteers that received the symbiotic shake exhibited a reduction in total cholesterol, n-HDL-C and triglycerides levels (25.84%, 41.30%; and 37.27%, respectively) and an increase on HDL-C concentrations (35.15%). Similar findings were reported by Larskin et al [15], who observed a reduction in total cholesterol and triglycerides after 5 weeks ingestion of *L. acidophilus *and *B. bifidum*. However, Lewis et al [21] did not observe any change in blood lipids in a double-blind, placebo-controlled study investigating the effect of yogurt with *Lactobacilllus acidophilus *on plasma lipid levels. De Roos et al [22] also did not observe any significant influence on the serum cholesterol of individuals who had consumed yogurt enriched with *Lactobacillus acidophilus*, though they did find statistically significantly higher HDL cholesterol concentrations in these same individuals. Similar results were observed by Kiebling et al [23], who reported a significant increase in HDL cholesterol with the administration of yogurt fermented by *L. acidophilus *145 and *B. logum *913 and added with 1% oligofructose. However, Greany et al [24], investigating capsules with combination of culture pure of *L. acidophilus *DDS-1, *B. logum *UABL-14 and FOS, did not observe any significant elevation in HDL.

Although many studies demonstrate the positive effect of prebiotics and probiotics on the modulation of serum cholesterol levels [25,26], some of these studies report conflicting findings and the mechanisms underlying the modulation of serum cholesterol levels by symbiotics remain largely obscure. According to Lee et al [27], five mechanisms may be involved in the reduction of cholesterol levels by lactic bacteria: (a) inhibition of the enzymatic synthesis of cholesterol by the fermentation products of lactic bacteria, mainly short chain fatty acids; (b) the bacteria may facilitate elimination of cholesterol through the feces; (c) inhibition of cholesterol absorption back into the body by binding with cholesterol; (d) the bacteria may interfere with the recycling of bile salts, facilitating their elimination. In this situation, cholesterol is mobilized to produce bile salts, resulting in a fall in blood cholesterol levels; (e) assimilation by lactic acid.

According Ooi and Liong [28] some species of Lactobacillus, Bifidobacterium, Enterococcus, and Streptococcus have demonstrated to have the capacity of lowering plasma cholesterol level. One of the underlying mechanisms is that these probiotic bacteria can reduce the reabsorption of bile acids through enterohepatic circulation. This is due to the fact that conjugated bile acids but not free forms are partly absorbed and directed back to the liver. Live lactobacilli and bifidobacteria cells can hydrolyze the conjugated bile acids, excrete them faster and reduce the extent to which they are reabsorbed.

In vitro experiments showed that probiotic bacteria, including *L. acidophilus *spp. and *B. bifidus *may assimilate cholesterol and deconjugate bile salts. The probiotic bacteria may assimilate dietary cholesterol, reducing absorption and increase bile excretion [28]. However, these effects are dependent on the ingestion of bacterial strains capable of colonizing the small intestine where cholesterol is absorbed [29].

In an earlier study it was evaluate the effect of the same symbiotic product on fecal microbiota of elderly people. The obtained results showed an increase in the population of *Bifidobacterium *spp. and *Lactobacillos *spp. (*P *< 0.01) and a decrease on *Clostridium *spp. and *Bacteroides *spp. (*P *< 0.05) counts [16]. The modification of the fecal microbiota composition observed during the ingestion of the synbiotic shake could collaborate to improve the lipid profile.

The effect of symbiotics on glycemia has not yet been fully elucidated and the data available on this subject are often somewhat contradictory, since they depend to a large extent on the specific physiological conditions of each individual. It is possible that, as is the case of other fibers, fructooligosaccharides influence the absorption of macronutrients, particularly carbohydrates, retard gastric emptying and/or decrease small-bowel transit time. Gluconeogenesis induced by inulin and oligofructose may be mediated by short-chain fatty acids, particularly propionate [26]. In our study, we observed a significant reduction in glycemia in the group that consumed the symbiotic shake. The findings of our study are in accordance with previously published data and reports in literature [30]. According to Luo et al [20] healthy volunteers who consumed 20 g fructooligosaccharides for 4 weeks presented a reduction of basal hepatic glucose production. In a clinical study conducted by Cicek et al [2], diabetes type 2 volunteers who consumed 20 g oligofructose per day exhibited a significant reduction in blood glucose levels after 6 weeks. According to Kaur & Gupta [31], inulin and oligofructose modulate the hormonal level of insulin and also the metabolism of carbohydrates and lipids by their ability to lower blood sugar levels.

In our experiments, we observed a numerical reduction in total cholesterol, a significant increase in HDL and a significant decrease in glycemia in the volunteers that ingested 200 mL/day of the symbiotic shake. No significant change was observed in the volunteers that took the placebo shake. A comparison between the results of our study with findings reported in the literature reveals a considerable number of discrepancies. According to Manzoni et al [32], these discrepancies may be due to the microorganism used, the volunteer population selected for the study, the manner in which the probiotic is administered and the duration of the protocol, with the positive effects being more evident in long-term protocols.

It should be noted that this study had some limitations that will be considered in future studies. First, the study population at the end of the protocol was small, since some volunteers had health problems that impeded the continuation of the study. The second limitation refers to the fact that volunteers make use of oral hypoglycemic agents and thus, the positive effects on blood glucose levels cannot be attributed only to the symbiotic product. However, since drug treatment was not able to maintain blood glucose of the study population at normal levels, could be attributed to the symbiotic shake a synergistic effect. Finally, the authors consider important the quantification of short-chain fatty acids produced by intestinal microbiota, using in vitro models, to relate to lipid-lowering effects observed.

## Conclusion

Based on the results obtained, it can be concluded that regular consumption of 200 mL of symbiotic shake for 30 days resulted in a positive change in the lipid profile and blood glucose level of the volunteers, and this product could be used to assist in maintaining lipids and glucose normal levels in the elderly population. Nonetheless, more studies will be required to confirm the effectiveness of the product.

## Abbreviations

TC: Total cholesterol; HDL-C: High density lipoprotein cholesterol; LDL-C: Low density lipoprotein cholesterol; VLDL: Very low density lipoprotein cholesterol; n-DHL-C: Non high density lipoprotein cholesterol; SCFA: Short-chain fatty acids.

## Competing interests

All authors have read and approved the final manuscript. All authors of this research have no conflict of interest related with employment, consultancies, stock ownership, honoraria, paid expert testimony, patent applications/registrations, and grants or other funding.

## Authors' contributions

Each author has participated sufficiently, intellectually or practically, in the work to take public responsibility for the content of the article, including the conception, design, and conduction of the experiment and for data interpretation (authorship). MC, ML, DCUC carried out the studies, sample analysis, data analyses, performed the statistical analysis and helped to draft the manuscript. SK participated in the design and coordination of the study, carried out the studies, and helped to draft the manuscript. CM participated in the design of the study and carried out the studies. All authors read and approved the final manuscript.
